# Decavanadate Toxicology and Pharmacological Activities: V_10_ or V_1_, Both or None?

**DOI:** 10.1155/2016/6103457

**Published:** 2016-01-21

**Authors:** M. Aureliano

**Affiliations:** ^1^Faculty of Sciences and Technology, University of Algarve, Campus of Gambelas, 8005-135 Faro, Portugal; ^2^CCMar (Centre of Marine Sciences), University of Algarve, Campus of Gambelas, 8005-135 Faro, Portugal

## Abstract

This review covers recent advances in the understanding of decavanadate toxicology and pharmacological applications. Toxicological* in vivo* studies point out that V_10_ induces several changes in several oxidative stress parameters, different from the ones observed for vanadate (V_1_). In* in vitro* studies with mitochondria, a particularly potent V_10_ effect, in comparison with V_1_, was observed in the mitochondrial depolarization (IC_50_ = 40 nM) and oxygen consumption (99 nM). It is suggested that mitochondrial membrane depolarization is a key event in decavanadate induction of necrotic cardiomyocytes death. Furthermore, only decavanadate species and not V_1_ potently inhibited myosin ATPase activity stimulated by actin (IC_50_ = 0.75 *μ*M) whereas exhibiting lower inhibition activities for Ca^2+^-ATPase activity (15 *μ*M) and actin polymerization (17 *μ*M). Because both calcium pump and actin decavanadate interactions lead to its stabilization, it is likely that V_10_ interacts at specific locations with these proteins that protect against hydrolysis but, on the other hand, it may induce V_10_ reduction to oxidovanadium(IV). Putting it all together, it is suggested that the pharmacological applications of V_10_ species and compounds whose mechanism of action is still to be clarified might involve besides V_10_ and V_1_ also vanadium(IV) species.

## 1. Introduction

The number of review papers and chapters reporting decavanadate biochemistry and biological activities has clearly increased since 2005 [1–5]. Besides the biological activities, decavanadate (V_10_O_28_^6−^) as well as other polyoxometalates (POMs) has a wide range of environmental, chemical, and industrial uses and applications in catalysis, nanomaterials, prevention of corrosion, smart glasses, macromolecular crystallography, and food chemistry, among others [6–9].

Decavanadate (V_10_) species are usually not taken into account in vanadium toxicological studies, although they are well known to affect the activity of several enzymes and to impact lipidic structures [1]. Besides,* in vivo* decavanadate toxicological studies remain seldom [1, 10]. One eventual reason is the consideration that almost 98% of vanadium in cells is present as oxidovanadium(IV), also known as vanadyl (+4 oxidation state), being the intracellular concentration of vanadium (+5, vanadate) very low to decavanadate species be formed. Previously, it was described that V_10_ was formed in acidic compartments in* Saccharomyces cerevisiae* that were grown in media containing vanadate [11]. It has been proposed that once formed the rate of decavanadate decomposition is slow (half-life time of hours) enough to allow observing its effects not only* in vitro* [12], but also* in vivo* [1, 10]. Furthermore, it was suggested that decameric vanadate can be stabilized upon interaction with cytoskeleton and membrane proteins and therefore its contribution to vanadium biochemistry and pharmacological activities can be enlarged [13]. For instance, it was described that rat adipocytes accumulate much more glucose upon decavanadate incubation than with known insulin mimetic agents such as bis(maltolato)oxovanadium(IV) (BMOV) [14]. Besides the insulin mimetic behavior, decavanadate and recent decavanadate compounds show several pharmacological activities such as anticancer, antibacterial, and antivirus [2, 15–17]. These recent findings, which are now briefly reviewed, are evaluated and several hypotheses and V_10_ modes of action through oxidative stress, effects in mitochondria, sarcoplasmic reticulum, and cytoskeleton, among other biological and pharmacological activities are analyzed.

## 2. Decavanadate and Oxidative Stress

In the last years, our research group has performed novel* in vivo* studies with decavanadate in order to understand the contribution of decameric vanadate species to vanadium toxic effects [1, 10]. First, at the specific experimental conditions, it was confirmed, using spectroscopy methodologies, if decavanadate is, or not, completely disintegrated into vanadate before inducing changes in several stress markers [1]. Secondly, following decavanadate solutions* in vivo* administration it was evaluated and also compared with monomeric vanadate solutions, several oxidative stress parameters, namely, reduced GSH content, overall rate of ROS production, lipid peroxidation, and antioxidant enzyme activities [1, 10].

First of all, it was concluded that the effects induced by both vanadate and decavanadate depend not only on the concentration but also on other experimental parameters such as the exposure time, cellular fraction, type of tissue, mode of administration, and species of animal [1, 3, 10]. Secondly, in the majority of the studies decavanadate clearly induced more, different, and, in many times, opposite effects than the ones observed for vanadate [3]. Thirdly, oxidative stress induced by decavanadate may be also due to decavanadate decomposition into vanadate [1, 3]. For instance, it was observed that the increase in GSH content upon decavanadate exposure was observed in experimental conditions where V_10_ is almost totally decomposed. The same suggestion was made for the increase in ROS production, with vanadate causing a larger increase in the first hour (150%) whereas decavanadate only caused also an increase (80%) after 12 hours, probably after dissociation into monomeric species [3].

It is known that the cellular detoxification mechanism proposed for vanadate involves bioreduction of vanadate to vanadyl by glutathione (GSH) [18]. Therefore, GSH is an important cellular antioxidant defense system and directly or indirectly regulates the levels of ROS [19, 20]. However, it is proposed that the mechanism for decavanadate detoxification is not the same, as it was suggested for the mechanism of thiol compounds oxidation by similar POMs [21]. Eventually, vanadate reduction by GSH may be delayed if decavanadate species are present. Hence, putative differences in the reactivity towards GSH may explain, at least in part, the different effects that vanadate and decavanadate solutions have in GSH levels and in ROS production. In the Fenton-like reactions vanadate is reduced to vanadyl with production of O_2_^•−^ [22]. It is possible that decavanadate participates in such reactions as well as in the GSH oxidation in a different manner and/or extension. On the other hand, lipid peroxidation is commonly described as a consequence of oxidative damage caused by ROS [19, 23]. It was described that lipid peroxidation propagation increased by 55% and 80% after 12 and 24 hours, respectively, in liver mitochondria on exposure to vanadate [1, 3] whereas no increase was evident after 12 hours in the case of decavanadate exposure. However, after 24 hours the effect induced by the latter was the same as that of vanadate [1, 3]. Similar oxidative stress behavior has been described in cardiac tissue [3], confirming that decavanadate seems to have a delayed effect on lipid peroxidation probably due to its decomposition into vanadate. Furthermore, for longer periods after exposure (seven days), decavanadate clearly differs from vanadate once it keeps the levels of lipid peroxidation high [3]. Regarding the antioxidants enzymes, it was suggested globally that decavanadate exposition induces a decrease in mitochondrial antioxidant enzymes activities such as SOD and catalase activities, whereas opposite or no effects were observed for vanadate [3]. Therefore, it is suggested that decavanadate species exposure follows different pathways than vanadate for the generation of reactive oxygen species and interferes differently with some of the enzymes involved in antioxidant defenses in cells. Besides, decavanadate slow decomposition would also induced delayed oxidative stress responses through vanadate species.

## 3. Mitochondria and Decavanadate Toxicity

Vanadium is a pollutant, and its toxic mechanisms are related to the production of oxidative stress [24]. Mitochondria provide the majority of the energy produced by aerobic organisms and are also often referred to as a major ROS production site. Therefore, mitochondria are a key issue for decavanadate toxicity and a tool to evaluate changes in several oxidative stress parameters, as described in the above section. Several studies pointed out mitochondria as a potential target for vanadium [25, 26] and variety of vanadium compounds, that is, vanadyl sulphate (VOSO_4_), sodium metavanadate (NaVO_3_), and vanadyl complexes with organic ligands [27]. Regarding decavanadate* in vivo* studies, it was suggested that the mitochondrial fraction tends to accumulate more vanadium upon decavanadate than upon vanadate administration, besides inducing different changes in mitochondrial antioxidant enzymes activities [1, 3]. This observation was further explored and* in vitro* studies were performed using cardiac mitochondria [28]. These studies showed that decavanadate inhibits mitochondrial respiration and induces mitochondrial membrane depolarization at nM range of decavanadate concentrations (IC_50_ values 40–100 nM) [28, 29]. Decavanadate effects on mitochondrial membrane depolarization and oxygen consumption are about hundredfold more strongly than monomeric vanadate [28]. The heart mitochondria from the fish (*Sparus aurata*) have been shown to be less sensitive to decavanadate than rat heart mitochondria, with IC_50_ values for decavanadate towards membrane depolarization and oxygen consumption that were about four times higher (196 and 400 nM, resp.) than the values found in the rat mitochondria studies (39 and 99 nM, resp.) [3, 28]. One the other side, *μ*M range of vanadate concentration is needed to induce the same effects: IC_50_ of 25 *μ*M and 50 *μ*M, respectively, for instance, for fish heart mitochondria. The effects induced by decavanadate are not due to the uncoupling of the mitochondria or associated with the mitochondrial permeability transition pore (MPTP), once the respiratory rate control was not changed or the inhibitor cyclosporine did not prevent effects induced upon decavanadate incubation.

**Figure 1:**
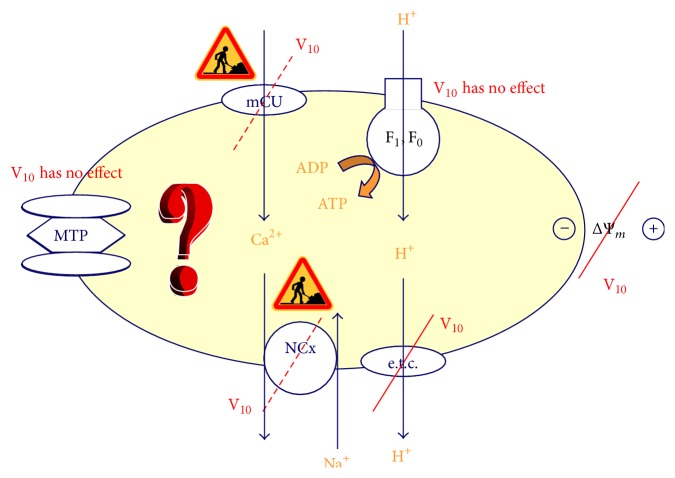
Mitochondria: a target for decavanadate (V_10_). Oxygen consumption and membrane depolarization are strongly affected whereas no effects were found for MTP and ATP synthase. To our knowledge, V_10_ effects on Ca^2+^ uniporter and Na^+^/Ca^2+^ exchanger were not yet described. F_1_F_0_, F_1_F_0_-ATP synthase; e.t.c., electron transport chain; mCU, Ca^2+^ uniporter; NCx, Na^+^/Ca^2+^ exchanger; MTP, mitochondrial transition pore; Ψ_*m*_, membrane potential.

Once the hypothesis that V_10_ affects respiratory complexes I and II is excluded, we considered the possibility that decameric vanadate may interfere with complex III (ubiquinol : cytochrome *c* oxidoreductase). The observed changes in the absorbance at 500–550 nm (0.05 OD increases) upon incubation with V_10_ pointed out that the cytochrome *b* redox state is altered and suggested that complex III is shifted to a more oxidized steady-state. Thus, decavanadate (20 *μ*M total vanadium concentration, i.e., decavanadate) affects the redox state of complex III cytochrome *b*, similarly as the well-known respiratory inhibitor antimycin-A [3, 28, 29]. With a similar total concentration of vanadate (20 *μ*M) no effect was observed [28, 29]. Notice that the respiratory rate control was not changed for either rat or fish heart mitochondria in the presence of either vanadium solution (5.0 ± 0.1) nor did the ADP/O values for pyruvate or malate; the vanadate solutions do not induce uncoupling of the mitochondria [28]. Furthermore, 2 *μ*M decavanadate did not affect NADH content, FoF1-ATPase, and cytochrome *c* oxidase, nor did it affect respiratory complexes I and II, pointing out to a specific V_10_ interaction with complex III (cytochrome *b*) from mitochondrial respiratory chain [3, 28]. The V_10_ effects can be summarized in the scheme present at Figure 1. By inducing mitochondria membrane depolarization and/or by inhibiting mitochondria respiratory chain, it is expected that V_10_ prevents the production of anion superoxide. In fact, both V_10_-induced mitochondrial depolarization and a decrease of mitochondrial superoxide anion generation can themselves account for a V_10_ antioxidant effect. A potential role for decavanadate in accepting electrons instead of oxygen is suggested. Conversely, V_10_ interaction with complex III would induce a leakage of electrons to molecular oxygen; it would be expected to induce the production of ROS.

Recently, the formation of vanadyl species upon decavanadate incubation with mitochondria (unpublished results), as analyzed by EPR, was observed, whereas no signals were detected upon vanadate incubation. It has been described previously that decavanadate may interact with NADH oxidation by oxygen catalyzed by several enzymes present in membrane systems such as the plasma membrane of red blood cells or rat liver microsomes that leads to reduction of cytochrome *c* [30] and to vanadate reduction. In other studies, decavanadate has been described to be reduced by a specific isocitrate dehydrogenase pointing out to a redox role for decavanadate [31]. These studies suggest an increasing of NADH oxidation by decavanadate, consequent oxygen reduction to H_2_O_2_, and concomitant reduction to vanadyl species [30, 31].

## 4. Decavanadate Toxicity Induces Apoptosis or Necrosis?

Vanadium causes a variety of toxic effects such as hematological and biochemical changes [10, 32]. Several studies have shown vanadate effects varying from stimulation of cell growth to induction of cell death [3, 20, 24, 32, 33]. In most cases, the vanadate effect on cell proliferation was biphasic, being cytotoxic for cells over a concentration range of 50 to 100 *μ*M [34]. By targeting mitochondria decavanadate might induce directly or indirectly processes of cell death. Besides, the inhibition of the mitochondrial respiratory chain described above by decavanadate can lead to sustained mitochondrial depolarization which is lethal for cells demanding a high supply of metabolic energy. Mitochondria are well-known organelle responsible for many features and processes of cell death, such as apoptosis and necrosis, and calcium homeostasis. Cell death is of course a parameter of toxicity and therefore questions arise: did the decavanadate toxicity effects induce cell death? In which way? The answer can be found on the studies that described that, upon incubation for 24 h with either decavanadate or vanadate, the concentrations were found to produce 50% loss of cell viability (1 *μ*M V_10_, and 10 *μ*M, resp.). Both vanadate species induce cardiomyocytes necrotic cell death, whereas no significant caspase-3 activation was observed [29]. It was also observed that the concentration needed for 50% mitochondrial depolarization was 0.65 *μ*M for V_10_ and 6.5 *μ*M for V_1_, that is, only slightly lower than the value obtained for vanadate induced 50% loss of cell viability [29]. Furthermore, depolarization of mitochondria was clearly observed even from 6 hours after addition of decavanadate to cardiomyocytes, suggesting a leading role of mitochondrial depolarization in V_10_-induced cardiomyocytes death and pointing out as an early event in decavanadate induced necrotic cell death of cardiomyocytes.

It is known that mitochondrial membrane depolarization leads to mitochondrial calcium release [35] and also IP_3_-mediated endoplasmic reticulum release in cardiomyocytes [36]. In fact, it was observed that the incubation of both decavanadate and vanadate with cardiomyocytes induces a rise of the basal cytosolic Ca^2+^ from 60 ± 10 nM to 200–250 nM, upon 24 h incubation with 1 *μ*M V_10_ or 10 *μ*M V_1_ [29]. These results are in agreement with earlier studies showing that vanadate increased intracellular Ca^2+^ in cultured aortic smooth muscle cells, thereby affecting the vascular tone [37]. In the heart, the release of Ca^2+^ from intracellular stores leads to an increase of heart rate and cardiac inotropism and to vasodilatation [36–38]. Thus, it is strongly suggested that mitochondrial membrane depolarization is a key event in decavanadate induced cardiomyocytes death. As referred to above, the effects described for decavanadate, after 24 hours of incubation, may be due to vanadate upon decavanadate slow decomposition.

## 5. Sarcoplasmic Reticulum and Decavanadate

Sarcoplasmic reticulum (SR) plays a crucial role in calcium homeostasis and in regulating the process of muscle contraction. SR Ca^2+^-ATPase is known to be responsible for actively transporting calcium ion, at ATP expenses, into SR lumen, and it plays a major role in the muscle relaxation process. The high sensitivity of the sarco/endoplasmic Ca^2+^-pumps to vanadate is well documented [39] providing also a simple explanation for the sustained rise of basal cytosolic Ca^2+^ concentration after incubation with vanadate and decavanadate solutions, as described above. However, it was demonstrated more than twenty years ago that decameric vanadate has specific features and interactions with SR Ca^2+^-ATPase, for instance, by inducing protein crystallization [40]. Besides, it was described that only decavanadate is able to inhibit SR Ca^2+^-ATPase calcium uptake, whereas no effects were observed for V_1_ [41]. Using several different methodologies, it was suggested that decavanadate interaction with the Ca^2+^-ATPase is noncompetitive versus ATP and that it inhibits strongly the ATPase activity (IC_50_ = 15 *μ*M), in comparison with V_1_ (IC_50_ = 80 *μ*M) [12, 39]. In the absence or in the presence of the natural ligand ATP, the interaction of V_10_ with the pump induces vanadate reduction, as analyzed using EPR spectroscopy [42]. During these studies, protein cysteine oxidation was detected upon V_10_ incubation, suggesting the involvement of cysteines at the V_10_ binding site as well as the participating of vanadyl species on the process of enzymatic inhibition. It is well established that the V_10_ binding site, which is formed by three proteins domains [43], is located at the cell cytoplasm side. V_10_ can interact with proteins by electrostatic interaction or by hydrogen bonding but the specific residues involved in V_10_-SR Ca^2+^-ATPase interaction, perhaps a cysteine residue, are yet to be totally clarified.

Once decavanadate binding site is located at the cytoplasmic site, V_10_ species would need to cross the SR membrane in order to bind to the E1E2 ATPase. Whereas the interaction between V_1_ and the E1E2 ATPase is only favored by the E2 conformation, V_10_ binds with all the two conformations E1 and E2, been or not phosphorylated, thus interacting also with E1P and E2P [12]. Therefore, it is suggested that decavanadate can affect all the steps of the mechanism of calcium translocation by the E1E2 ATPase. Perhaps due to this particularity, V_10_ interaction with the ion pumps might also occur through the extracellular side, as is the case with several other drugs that impact these proteins [3]. By targeting ion pumps without needing to cross the membrane, decavanadate can more rapidly induce changes in calcium homeostasis with implications in, for example, muscle contraction, calcium accumulation in mitochondria, and concomitantly ROS production and cell death.

Studies with the SR Ca^2+^-ATPase were also performed upon decavanadate* in vivo* intravenous administration [1, 3]. Thus, measurements of the skeletal muscle SR Ca^2+^-ATPase activity, performed 48 hours upon administration of decavanadate (70.4 ± 6.65 nmol Pi/min/mg), showed that the ATP hydrolysis is significantly increased (52%), whereas vanadate solution exposure decreased it by 15%. These results seem in opposition to previously decavanadate and vanadate inhibition studies performed* in vitro* with the SR Ca^2+^-ATPase pump. It is difficult to explain why the sarcoplasmic reticulum vesicles prepared from animals previously exposed to V_10_ present higher ATPase activity and are opposite to the ones observed for vanadate. Vanadate is known for its ability to increase the contractile force of heart muscle through its inotropic effect [37, 38, 44]. Apparently, V_10_ affects differently the calcium homeostasis and the samples used contain more calcium or V_10_ interaction would induce the formation of ATPase dimers, eventually relevant for ATPase activity. These very interesting observations that need further studies point out that different responses obtained upon* in vivo* administration cannot be always associated with* in vitro* studies and prove that great care must be taken with extrapolations from* in vitro* to* in vivo* conditions and vice versa.

## 6. Decavanadate and Muscle Contraction

Several recent review papers clearly point out that decavanadate presents many biological activities affecting several biological processes and biochemical mechanism including the mechanism of muscle contraction and its regulation [1, 3]. Skeletal muscle cells and vanadium are historically strongly connected to each other, since vanadium was identified as an impurity in commercial ATP prepared from equine muscle [45]. However, the essentiality of vanadium in muscle and globally in humans is yet to be clarified [46]. Myosin, the major ATPase of muscle, interacts with actin during the process of muscle contraction. Although some aspects are poorly understood, during the contractile cycle, the rate limiting step of the ATP hydrolysis is the release of Pi from myosin, which is accelerated by the rebinding of actin [47]. It has originally demonstrated that, in the absence of actin, vanadate inhibits myosin ATPase activity [48]. However, only decavanadate inhibits myosin ATPase activity stimulated by actin [49]. A simple mechanism for the experimentally observed noncompetitive inhibition pattern of V_10_ towards both ATP and actin, as it does not interfere with the nucleotide binding site or with actin binding surface, is by acting as a “back-door” blocking the actomyosin cycle, most likely, in the prehydrolysis state [1, 49, 50].

When we compare the effects of decavanadate on myosin ATPase, Ca^2+^-ATPase, actin polymerization, and myosin ATPase activity stimulated by actin, the latter presented the higher decavanadate inhibitory capacity with an IC_50_ value of 0.75 *μ*M, whereas higher inhibitory IC_50_ values were found for Ca^2+^-ATPase activity (15 *μ*M) and for actin polymerization (68 *μ*M) [3, 12, 13, 49]. It was suggested that skeletal muscle myosin contains a high affinity decavanadate binding site, being a potential target for decavanadate [49].

Recent studies also described a specific decavanadate interaction with the actin monomer, G-actin, at the ATP binding site [3, 50]. Actin is one of the most abundant proteins in cells, being involved in many cellular and biological processes. It has been described that vanadium induces actin cytoskeleton damage associated with impaired fertility [51]. However, the studies about “vanadium and actin,” and more specifically with “decavanadate and actin,” remain scarce [1, 3, 50]. As it was above described for the V_10_-SR Ca^2+^-ATPase interaction, also the decavanadate interaction with G-actin leads to cysteine oxidation and vanadyl formation, whereas no reductions were observed upon vanadate incubation [1, 3]. In contrast to the calcium pump, ATP prevents the formation of vanadyl species, confirming that V_10_ binds to the ATP binding site. Both decavanadate and vanadyl inhibit actin polymerization. It was further observed that actin contains a high affinity binding site for vanadyl, as it happens with other proteins such as transferrin and albumin. Therefore, it is suggested that, in the absence of ATP, decavanadate interactions with actin lead to vanadyl protein binding, although the mechanism by which decavanadate inhibits actin polymerization, for instance, through vanadyl formation, is yet to be clarified.

## 7. Decavanadate Pharmacological Activities

The majority of the studies described above support the concerns over the potential risk of the use of vanadyl sulphate in athletes as a sport supplement [44]. In fact, once vanadium is slowly eliminated from mammalian tissues [52], chronic consumption of vanadium compounds, such as vanadyl sulphate, may eventually reach the toxic levels to cardiomyocytes. Furthermore, decavanadate, vanadate, or vanadyl interferes, although differently, with muscle proteins and with the process of muscle contraction and its regulation. The processes by which vanadyl compounds, commonly used by the bodybuilders, increase muscle mass and enhance muscle power are not understood. In fact, the biochemical processes involved whether being related with muscle bioenergetics, metabolism, and functionality of the contractile systems or through the increasing of the muscle fibers is almost completely unknown. The role of vanadium in muscle cells and its essentiality to humans still remains a mystery.

Regarding the use of vanadate species as anticancer agents and in the chemotherapy of multidrug-resistant tumors, also the studies described above might be in contradiction with this possibility due to its toxic effects, being manifested even at very low concentrations. Nevertheless, as described briefly below, the pharmacological activities of decavanadate, and decavanadate compounds as antidiabetic, antivirus, antibacterial, and antitumor agents, is actually a matter of increasing interest.

For V_10_ alone, it was reported that rat adipocytes incubated with decavanadate at 37°C, thus favoring V_10_ decomposition, accumulate much more glucose than with other known insulin mimetic agents such as BMOV or vanadate [13]. It is suggested that the agents (enzymes, receptors, pumps, or channels) involved in the early events of the process of glucose transport can be enhanced and/or potentiated by V_10_. However, to our knowledge, the V_10_ mechanism or contribution as an insulin mimetic agent or enhancer is yet to be totally clarified. Eventually, as it was referred for vanadate, decavanadate insulin mimetic effects are probably induced through the inhibition of tyrosine phosphatase (PTP) [53]. Moreover, it was speculated that V_10_ could have a role in treating* Leishmania* diseases through PTP inhibition [53]. Another mechanism includes the use of decavanadate compounds as a prodrug of peroxovanadate insulin mimetics [54]. Crystallization of decavanadate in a spatially selective manner within the protein cages of virions is the most cited paper regarding V_10_ in biology [17]. As it was described in a fundamental review, the antiviral and antitumor activities are the dominant activities of POMs in pharmacology and medicine [55]. It seems that POMs such as decavanadate are able to inhibit the virus activities by preventing the virus-cell host binding [56]. POMs low toxicity toward human body and their high solubility in water are main factors that contributed to their development as drugs.

A chitosan-decavanadate complex with antibacterial activity against* Escherichia coli* and* Staphylococcus aureus* was recently described [57]. Chitosan is famous for its antimicrobial activity as it inhibits the mRNA synthesis after penetration into the nuclei of the microorganism. On the other hand, decavanadate is known for its inhibition of ion pumps causing a disturbance in the molecular transport across the membrane thus devastating the bacteria metabolism, presenting altogether an antibacterial inhibition of 12.5 *μ*M. The antitumour activity of decavanadate is less understood and more recent than the antiviral one. New decavanadate complexes have been synthetized and tested their antitumor activity* in vitro* against human lung carcinoma cells (A549) and murine leukaemia cells (P388) [58]. Both compounds exhibited lower inhibition than cis-platin compounds, whereas the decavanadate compound with a higher lipophilic effect, thus enhancing its penetration through the lipid bilayer of the cell membrane, showed higher inhibitory activity [58]. The cytotoxicity of both V_10_ compounds was tested on human normal hepatocytes being more or equally toxic against normal cells compared to effective against cancer cells. Other decavanadate complexes were reported as antitumor agents, showing apoptotic mechanism of cell death and also lower activities than platin compounds [15, 16, 58].

Although the antitumor activity of V_10_ compounds against a large number of tumor cells has been reported, it looks as if their mechanisms of action are still difficult to understand. It was described that polyoxometalates are able to inhibit the tumor growth by inducing apoptosis. Some studies suggest that POMs entered into the mitochondrion leading to the inhibition of ATP synthesis [55]. Although it is speculated that V_10_ effects in mitochondria can be applied for other POMs, these studies are apparently in opposition with the studies described above regarding the process of cell death induced by decavanadate in cardiomyocytes.

Notice that, in the majority of the studies described above, the stability of decavanadate compounds, at the several experimental conditions, was not performed or takes in consideration its putative reduction or decomposition into vanadate species. As described above, although decavanadate toxicological and pharmacological applications differ from vanadate, we cannot exclude a participation of monomeric vanadate. Furthermore, decavanadate toxicity effects and pharmacological activities can be due, at least in part, to V_10_ reduction to vanadyl species. Therefore, although some decavanadate compounds have been shown to be stable, care must be taken before attributing them the toxicity effects or the pharmacological activities [1, 3].

It is known that abnormal levels of alkaline phosphatases (ALP) in the serum are detected in cancer patients since tumors are abnormal cellular growth proliferating faster than a normal cell. Inhibition of ALP will affect tumor cell metabolism and function and therefore POMs were assessed for their inhibitory effect on ALP activity and as putative antitumor agent [59]. V_10_ also demonstrated inhibition on several alkaline phosphatases, suggesting that decavanadate, similarly to other POMs, inhibits abnormal cellular growth proliferating. Despite promising results against virus, bacteria, and tumor cells, polyoxometalates and V_10_ are not yet tested in clinical trials. This may be due to the lack of understanding of its mechanism of action. Besides, to be approved as a drug the polyoxometalate or the V_10_ compounds must show higher activity against tumor cells and very low toxicity toward normal cells.

## 8. Conclusions

Oxidative stress induced by decavanadate would occur in organisms more often than expected. Decavanadate mechanisms to induce stress might involve the interaction with ion pumps, mitochondria, and specific biochemical processes. The mechanism of necrotic cell death induced by decavanadate is proposed to be mediated through mitochondrial membrane depolarization. The simultaneous effects in ion pumps and in mitochondria promoted by decavanadate lead to an intracellular calcium increase, changes in ROS producing, and inhibition on antioxidant enzymes activities, namely, SOD and catalase. Several major proteins in muscle contraction and its regulation are molecular targets for decavanadate. Particularly interesting is the proposed back-door mechanism of V_10_ myosin ATPase inhibition stimulated by actin and also the inhibition of actin polymerization by decavanadate, although the latter process is still to be clarified. Some decavanadate compounds seem not suited for antitumour activity since their cytotoxicity was higher than its inhibitory rate of tumor cell growth. However, decavanadate was used with success in antibacterial activity and described to present many other pharmacological applications such as antidiabetic agent besides against virus activities. Putting it all together, it is proposed that the understanding of decavanadate toxicology and pharmacological activities may be useful, at least in part, to elucidate the biological activities of several polyoxometalates in order to make them available and safe for clinical use.

## Abbreviations

ALP: Alkaline phosphatase
BMOV: Bis(maltolato)oxovanadium(IV)
GSH: Reduced glutathione
MPTP: Mitochondrial permeability transition pore
NaVO_3_: Sodium metavanadate
POMs: Polyoxometalates
PTP: Protein tyrosine phosphatase
ROS: Reactive oxygen species
SOD: Superoxide dismutase
SR: Sarcoplasmic reticulum
VOSO_4_: Vanadyl sulphate
V_1_: Vanadate, monomeric vanadate containing 1 vanadate unit
V_10_: Decavanadate, vanadate oligomer containing 10 vanadate units.

## References

[1] Aureliano M. (2011). Recent perspectives into biochemistry of decavanadate. *World Journal of Biological Chemistry*.

[2] Toumi S., Ratel-Ramond N., Akriche S. (2015). Decavanadate cage-like cluster templated by organic counter cation: synthesis, characterization and its antimicrobial effect against Gram positive *E. Feacium*. *Journal of Cluster Science*.

[3] Aureliano M., Ohlin C. A. (2014). Decavanadate in vitro and in vivo effects: facts and opinions. *Journal of Inorganic Biochemistry*.

[4] Hayashi Y. (2011). Hetero and lacunary polyoxovanadate chemistry: synthesis, reactivity and structural aspects. *Coordination Chemistry Reviews*.

[5] Chen X., Yan S., Wang H., Hu Z., Wang X., Huo M. (2015). Aerobic oxidation of starch catalyzed by isopolyoxovanadate Na_4_Co(H_2_O)_6_V_10_O_28_. *Carbohydrate Polymers*.

[6] Mohapatra L., Parida K. M. (2014). Dramatic activities of vanadate intercalated bismuth doped LDH for solar light photocatalysis. *Physical Chemistry Chemical Physics*.

[7] Bijelic A., Rompel A. (2015). The use of polyoxometalates in protein crystallography—an attempt to widen a well-known bottleneck. *Coordination Chemistry Reviews*.

[8] Chen B. N., Xing R., Wang F., Zheng A. P., Wang L. (2015). Inhibitory effects of *α*-Na8SiW11CoO40 on tyrosinase and its application in controlling browning of fresh-cut apples. *Food Chemistry*.

[9] Yerra S., Tripuramallu B. K., Das S. K. (2014). Decavanadate-based discrete compound and coordination polymer: synthesis, crystal structures, spectroscopy and nano-materials. *Polyhedron*.

[10] Borges G., Mendonça P., Joaquim N., Coucelo J. M., Aureliano M. (2003). Acute effects of vanadate oligomers on heart, kidney, and liver histology in the Lusitanian toadfish (*Halobatrachus didactylus*). *Archives of Environmental Contamination and Toxicology*.

[11] Willsky G. R., White D. A., McCabe B. C. (1984). Metabolism of added orthovanadate to vanadyl and high-molecular-weight vanadates by *Saccharomyces cerevisiae*. *The Journal of Biological Chemistry*.

[12] Fraqueza G., Ohlin C. A., Casey W. H., Aureliano M. (2012). Sarcoplasmic reticulum calcium ATPase interactions with decaniobate, decavanadate, vanadate, tungstate and molybdate. *Journal of Inorganic Biochemistry*.

[13] Ramos S., Manuel M., Tiago T. (2006). Decavanadate interactions with actin: inhibition of G-actin polymerization and stabilization of decameric vanadate. *Journal of Inorganic Biochemistry*.

[14] Pereira M. J., Carvalho E., Eriksson J. W., Crans D. C., Aureliano M. (2009). Effects of decavanadate and insulin enhancing vanadium compounds on glucose uptake in isolated rat adipocytes. *Journal of Inorganic Biochemistry*.

[15] Galani A., Tsitsias V., Stellas D., Psycharis V., Raptopoulou C. P., Karaliota A. (2015). Two novel compounds of vanadium and molybdenum with carnitine exhibiting potential pharmacological use. *Journal of Inorganic Biochemistry*.

[16] Kioseoglou E., Gabriel C., Petanidis S. (2013). Binary decavanadate-betaine composite materials of potential anticarcinogenic activity. *Zeitschrift fur Anorganische und Allgemeine Chemie*.

[17] Douglas T., Young M. (1998). Host–guest encapsulation of materials by assembled virus protein cages. *Nature*.

[18] Legrum W. (1986). The mode of reduction of vanadate(+V) to oxovanadium (+IV) by glutathione and cysteine. *Toxicology*.

[19] Capella L. S., Gefé M. R., Silva E. F. (2002). Mechanisms of vanadate-induced cellular toxicity: role of cellular glutathione and NADPH. *Archives of Biochemistry and Biophysics*.

[20] Zhang Z., Leonard S. S., Huang C., Vallyathan V., Castranova V., Shi X. (2003). Role of reactive oxygen species and MAPKs in vanadate-induced G(2)/M phase arrest. *Free Radical Biology and Medicine*.

[21] Chakrabarty S., Banerjee R. (2015). Kinetics and mechanism of oxidation of 2-mercaptoethanol by the heteropolyoxovanadate [MnV_13_O_38_]^7−^. *International Journal of Chemical Kinetics*.

[22] Stohs S. J., Bagchi D. (1995). Oxidative mechanisms in the toxicity of metal ions. *Free Radical Biology and Medicine*.

[23] Byczkowski J. Z., Kulkarni A. P., Nriagu J. O. (1998). Oxidative stress and pro-oxidant biological effects of vanadium. *Vanadium in the Environment, Part 1: Chemistry and Biochemistry*.

[24] Colin-Barenque L., Pedraza-Chaverri J., Medina-Campos O. (2015). Functional and morphological olfactory bulb modifications in mice after vanadium inhalation. *Toxicologic Pathology*.

[25] Edel J., Sabbioni E. (1993). Accumulation, distribution and form of vanadate in the tissues and organelles of the mussel *Mytilus edulis* and the goldfish *Carassius auratus*. *Science of the Total Environment*.

[26] Bracken W. M., Sharma R. P., Elsner Y. Y. (1985). Vanadium accumulation and subcellular distribution in relation to vanadate induced cytotoxicity in vitro. *Cell Biology and Toxicology*.

[27] Zhao Y., Ye L., Liu H. (2010). Vanadium compounds induced mitochondria permeability transition pore (PTP) opening related to oxidative stress. *Journal of Inorganic Biochemistry*.

[28] Soares S. S., Gutiérrez-Merino C., Aureliano M. (2007). Mitochondria as a target for decavanadate toxicity in *Sparus aurata* heart. *Aquatic Toxicology*.

[29] Soares S. S., Henao F., Aureliano M., Gutiérrez-Merino C. (2008). Vanadate induces necrotic death in neonatal rat cardiomyocytes through mitochondrial membrane depolarization. *Chemical Research in Toxicology*.

[30] Ramasarma T., Rao A. V. S. (2006). Decavanadate interacts with microsomal NADH oxidation system and enhances cytochrome c reduction. *Molecular and Cellular Biochemistry*.

[31] Rao A. V. S., Ramasarma T. (2000). NADH-dependent decavanadate reductase, an alternative activity of NADP-specific isocitrate dehydrogenase protein. *Biochimica et Biophysica Acta (BBA)—General Subjects*.

[32] Ghosh S. K., Saha R., Saha B. (2015). Toxicity of inorganic vanadium compounds. *Research on Chemical Intermediates*.

[33] Capella L. S., Alcantara J. S. M., Moura-Neto V., Lopes A. G., Capella M. A. M. (2000). Vanadate is toxic to adherent-growing multidrug-resistant cells. *Tumor Biology*.

[34] Etcheverry S. B., Cortizo A. N., Nriagu J. O. (1998). Bioactivity of vanadium compounds on cells in culture. *Vanadium in the Environment, Part 1: Chemistry and Biochemistry*.

[35] O'Reilly C. M., Fogarty K. E., Drummond R. M., Tuft R. A., Walsh J. V. (2004). Spontaneous mitochondrial depolarizations are independent of SR Ca^2+^ release. *American Journal of Physiology—Cell Physiology*.

[36] Poindexter B. J., Smith J. R., Buja L. M., Bick R. J. (2001). Calcium signalling mechanisms in dedifferentiated cardiac myocytes: comparison with neonatal and adult cardiomyocytes. *Cell Calcium*.

[37] Sandirasegarane L., Gopalakrishnan V. (1995). Vanadate increases cytosolic free calcium in rat aortic smooth muscle cells. *Life Sciences*.

[38] Braunwald E. (1994). Vanadate increases cytosolic free calcium in rat aortic smooth muscle cells. *Cardioscience*.

[39] Caroni P., Carafoli E. (1981). The Ca^2+^-pumping ATPase of heart sarcolemma. Characterization, calmodulin dependence, and partial purification. *Journal of Biological Chemistry*.

[40] Dux L., Martonosi A. (1983). Two-dimensional arrays of protein in sarcoplasmic reticulum and purified Ca^2+^-ATPase vesicles treated with vanadate. *The Journal of Biological Chemistry*.

[41] Aureliano M., Madeira V. M. C. (1994). Interactions of vanadate oligomers with sarcoplasmic reticulum Ca^2+^-ATPase. *Biochimica et Biophysica Acta (BBA)—Molecular Cell Research*.

[42] Fraqueza G., Batista de Carvalho L. A. E., Marques M. P. M. (2012). Decavanadate, decaniobate, tungstate and molybdate interactions with sarcoplasmic reticulum Ca^2+^-ATPase: quercetin prevents cysteine oxidation by vanadate but does not reverse ATPase inhibition. *Dalton Transactions*.

[43] Hua S., Inesi G., Toyoshima C. (2000). Distinct topologies of mono- and decavanadate binding and photo-oxidative cleavage in the sarcoplasmic reticulum ATPase. *The Journal of Biological Chemistry*.

[44] Fawcett J. P., Farquhar S. J., Walker R. J., Thou T., Lowe G., Goulding A. (1996). The effect of oral vanadyl sulphate on body composition and performance in weight-training athletes. *International Journal of Sport Nutrition*.

[45] Josephson L., Cantley L. C. (1977). Isolation of a potent (Na-K)ATPase inhibitor from striated muscle. *Biochemistry*.

[46] Gruzewska K., Michno A., Pawelczyk T., Bielarczyk H. (2014). Essentiality and toxicity of vanadium supplements in health and pathology. *Journal of Physiology and Pharmacology*.

[47] Månsson A., Rassier D., Tsiavaliaris G. (2015). Poorly understood aspects of striated muscle contraction. *BioMed Research International*.

[48] Goodno C. C. (1979). Inhibition of myosin ATPase by vanadate ion. *Proceedings of the National Academy of Sciences of the United States of America*.

[49] Tiago T., Aureliano M., Gutiérrez-Merino C. (2004). Decavanadate binding to a high affinity site near the myosin catalytic centre inhibits F-actin-stimulated myosin ATPase activity. *Biochemistry*.

[50] Ramos S., Duarte R. O., Moura J. J. G., Aureliano M. (2009). Decavanadate interactions with actin: cysteine oxidation and vanadyl formation. *Dalton Transactions*.

[51] Rodriguez-Lara V., Morales-Rivero A., Rivera-Cambas A. M., Fortoul T. I. (2013). Vanadium inhalation induces actin changes in mice testicular cells. *Toxicology and Industrial Health*.

[52] Barceloux D. G. (1999). Vanadium. *Journal of Toxicology: Clinical Toxicology*.

[53] Turner T. L., Nguyen V. H., McLauchlan C. C. (2012). Inhibitory effects of decavanadate on several enzymes and *Leishmania tarentolae* in vitro. *Journal of Inorganic Biochemistry*.

[54] Yraola F., Garcia-Vicente S., Marti L., Albericio F., Zorzano A., Royo M. (2007). Understanding the mechanism of action of the novel SSAO substrate (C_7_NH_10_)_6_(V_10_O_28_)·2H_2_O, a prodrug of peroxovanadate insulin mimetics. *Chemical Biology and Drug Design*.

[55] Rhule J. T., Hill C. L., Judd D. A., Schinazi R. F. (1998). Polyoxometalates in medicine. *Chemical Reviews*.

[56] Yamase T. (2005). Anti-tumor, -viral, and -bacterial activities of polyoxometalates for realizing an inorganic drug. *Journal of Materials Chemistry*.

[57] Li Y.-T., Zhu C.-Y., Wu Z.-Y., Jiang M., Yan C.-W. (2010). Synthesis, crystal structures and anticancer activities of two decavanadate compounds. *Transition Metal Chemistry*.

[58] Zhai F., Wang X., Li D., Zhang H., Li R., Song L. (2009). Synthesis and biological evaluation of decavanadate Na_4_Co(H_2_O)_6_V_10_O_28_·18H_2_O. *Biomedicine & Pharmacotherapy*.

[59] Raza R., Matin A., Sarwar S. (2012). Polyoxometalates as potent and selective inhibitors of alkaline phosphatases with profound anticancer and amoebicidal activities. *Dalton Transactions*.

